# Elimination of Solanum nigrum ilarvirus 1 and Apple Hammerhead Viroid from Apple Cultivars Using Antivirals Ribavirin, Rimantadine, and Zidovudine

**DOI:** 10.3390/v15081684

**Published:** 2023-08-02

**Authors:** Jiří Sedlák, Jaroslava Přibylová, Igor Koloňuk, Josef Špak, Ondřej Lenz, Matěj Semerák

**Affiliations:** 1 Research and Breeding Institute of Pomology Holovousy, Ltd., Holovousy 129, 50801 Holovousy, Czech Republic; matej.semerak@vsuo.cz; 2 Czech Academy of Sciences, Biology Centre, Institute of Plant Molecular Biology, Branišovská 31, 37005 České Budějovice, Czech Republic; pribyl@umbr.cas.cz (J.P.); spak@umbr.cas.cz (J.Š.); lenz@umbr.cas.cz (O.L.)

**Keywords:** apple, virus, viroid, HTS, chemotherapy

## Abstract

Apple hammerhead viroid (AHVd) was detected in the apple cultivar ‘Šampion’ and in mixed infection with Solanum nigrum ilarvirus 1 (SnIV-1) in the cultivars ‘Selena’ and ‘Jonagored Supra’, using a high-throughput sequencing method. Experiments were conducted to eliminate both pathogens in apples using meristem tip cultures in combination with the antivirotics ribavirin, rimantadine, and zidovudine. Elimination of both pathogens was verified by repeated RT-PCR and qRT-PCR assays after 7–11 months. Elimination of SnIV-1 from all cultivars was successful with each of the three antivirotics at concentrations of 20, 40, and 80 mg L^−1^. Elimination of AHVd was also achieved, although less effectively and only with ribavirin in the concentration range of 20–160 mg L^−1^.

## 1. Introduction

Virus-like pathogens (viruses, viroids, and phytoplasmas) cause billions of euros in economic losses regarding the quality and yield of agricultural production worldwide. For some cultivated plants, these losses are so high that they threaten the very profitability of their own production and global food security [1,2,3]. The main cause of economically impactful crop epidemics is the international agricultural trade in seeds, vegetatively propagated planting material, and food products, which are transported in large quantities across and between continents [2,4]. Each crop is affected by a number of economically important virus-like pathogens. Apple cultivars grown in orchards can be infected by at least 21 known viruses and 8 viroids [5,6], and once infected, trees remain infected throughout their life. Preference should be given to prevention of infection rather than implementing costly measures for treatment or eradication. Early and reliable detection and identification of the causative agents are the basis for all protective measures and therefore are of considerable practical importance [7,8]. Rapid detection methods are also essential for understanding the biology and epidemiology of systemic plant infections. Due to the impossibility of using conventional plant protection products against virus-like pathogens, the production of healthy propagating plant material of fruit trees is a key preventive and protective measure within the certification system.

High-throughput sequencing (HTS) testing of agricultural crop varieties allows unbiased detection of the nucleic acid sequences of all virus-like pathogens [9]. Its use in practice represents the possibility of obtaining a completely new quality of propagation material of apple varieties and rootstocks. One of the objectives of our previous study [10] was to determine by HTS testing whether it is possible to find plants completely free of virus-like sequences among apple cultivars maintained as elite plant material in the insect -proof screenhouse of the Research and Breeding Institute of Pomology Holovousy Ltd. (RBIP). During this study, we detected apple hammerhead viroid (AHVd) [11] in the cultivar ‘Šampion’, and AHVd in mixed infection with Solanum nigrum ilarvirus 1 (SnIV-1) [12] in ‘Selena’ and ‘Jonagored Supra’ cultivars. Both AHVd- and SnIV-1-positive cultivars showed no specific symptoms on leaves or stems in either single or mixed infection scenarios. AHVd [13] and SnIV-1 [6] have only recently been found infecting apple trees.

‘Jonagored Supra’ is becoming an important commercial genotype grown worldwide. The original Czech cultivars ‘Šampion’ and ‘Selena’ excel in good taste and high productivity and have the potential for further expansion in the commercial Central European apple-growing sector. Therefore, the aim of this study was to make the first attempt to sanitise the cultivars’ primary stock material in the form of shoot tips infected with AHVd and SnIV-1 using the antivirals ribavirin, rimantadine, and zidovudine to allow for their certification as elite propagation material. Ribavirin, a nucleoside analogue with several putative mechanisms of antiviral activity [14], was used because it has been suggested as the most effective antiviral compound against plant viruses [15,16], and rimantadine and zidovudine were used because of promising results in recent studies [17,18]. As part of the experiment, the phytotoxicity of these antivirals was also tested.

## 2. Materials and Methods

The virus statuses of selected initial mother plants of the apple cultivars ‘Šampion’, ‘Jonagored Supra’, and ‘Selena’ maintained in technical isolation at the RBIP were evaluated before the beginning of chemotherapy by HTS [10]. The HTS results were validated with RT-(q)PCR.

**Figure 1:**
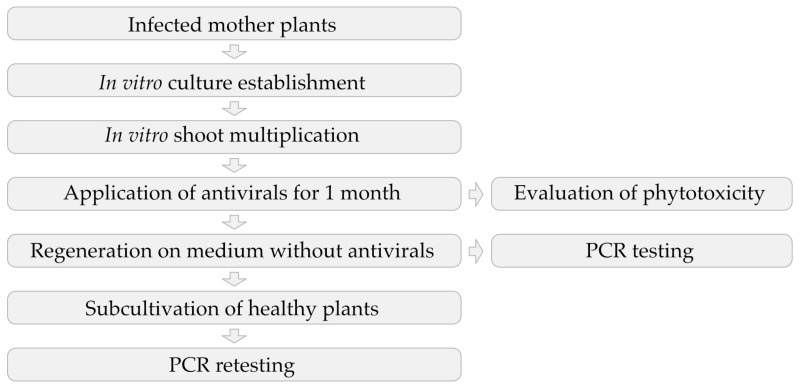
Scheme of in vitro chemotherapy with application of antivirals.

The whole procedure of experiment establishment, chemotherapeutical treatment, and PCR testing, described further, is depicted as a scheme (Figure 1).

### 2.1. In Vitro Cultures

Shoot cultures were established from 8–10 mm long excised tips taken from actively growing shoots of infected apple mother plants. The apical parts of shoots from three infected apple cultivars were prepared for the sterilisation procedure by removing all expanded leaves. The explants were sterilized in a 0.15% solution of mercuric chloride. Several drops of wetting agent (Tween-20) were added to the sterilization solution. The sterilization time was 1 min, followed by three rinses with sterile distilled water. All manipulations were performed in a sterile environment in a laminar flow hood.

The sterilized shoot tips were placed in 100 mL Erlenmeyer flasks containing 25 mL of culture medium and 5 explants each. The culture medium consisted of Murashige and Skoog (MS) salts and vitamins [19] supplemented with 100 mg L^−1^ inositol, 2 mg L^−1^ glycine, 30 g L^−1^ sucrose, 1.5 mg L^−1^ 6-benzylaminopurine (BAP), and 0.1 mg L^−1^ indole-3-butyric acid (IBA). The medium was gelled with 0.85% (*w*/*v*) Difco Bacto agar. The pH of the medium was adjusted to 5.7 before the agar was added and autoclaved at 120 °C for 15 min. All cultures were incubated in a growth room at 22 ± 1 °C and a 16 h photoperiod under cool white fluorescent light provided by Sylvania/Germany tubular lamps (F18W/840-TB) positioned 30 cm above the level of the cultures.

### 2.2. Antivirals

For our experiments, ribavirin (Duchefa Biochemie, The Netherlands) was selected as a broad-spectrum antiviral drug with a putative virostatic effect. Ribavirin (Virazole) is a synthetic guanosine nucleoside: 1-β-D-ribofuranosyl-1,2,4-triazole-3-carboxamide [20]. Its antiviral activity has been reported against a wide range of RNA and DNA zoonotic and human viruses [20,21]. Subsequently, ribavirin became more readily available, including as a generic drug, and was successfully used for chemotherapy of viruses infecting a wide range of plant species including apple [15,16,22].

Zidovudine (United States Pharmacopeia; also known as azidothymidine or AZT), is a synthetic analogue of 3’-azido-3’-deoxythymidine. It belongs to the class of antiretroviral nucleoside analogue reverse transcriptase inhibitors used in a complex with other antiviral drugs against HIV. HIV and other retroviruses, which consist of two single-stranded RNA strands, use reverse transcriptase to synthesize DNA when they enter a host cell. Zidovudine inhibits this enzyme, thereby reducing viral replication [23,24].

Rimantadine (Alfa Aesar, Tewksbury, MA, USA) is a cyclic amine 1-(adamantan-1-yl)ethan-1-amine discovered in 1963 [25], and it is used to treat influenza type A under the trade name Flumadine. It has been shown to inhibit the influenza proton channel M2 by binding into its pore [26]. When used against the hepatitis A virus, rimantadine application was shown to increase the number of lysosomes and their fusion with autophagosomes, which enhances autophagy [27].

### 2.3. Application of Antivirals

In vitro cultures were serially subcultured at four-week intervals for three months in MS medium supplemented with 1.5 mg L^−1^ BAP and 0.1 mg L^−1^ IBA. This provided a stock collection of shoots for chemotherapy treatment. The individual explants, in the form of single shoots with at least four well-developed leaves, were aseptically trimmed to a length of 8–10 mm using a scalpel. The shoot tips were then transferred to the treatment media with the antiviral agent. The antiviral compounds ribavirin, rimantadine, and zidovudine were filter-sterilised (Acrodisc Syringe Filter 0.2 µm, Pall Gelman, AZ, USA; diameter 25 mm) and added to reach final concentrations of 20, 40, 80, 160, 320, 640, and 1280 mg L^−1^ in the same MS medium as used for multiplication. The in vitro cultures of infected plants were grown on media with antivirals for four weeks under the same conditions as for multiplication. For each cultivar and antiviral compound concentration, a total of 10 in vitro initial shoot tips were grown and observed during and after the chemotherapy procedures to evaluate their survival and the potential phytotoxicity of the antiviral. After the treatment with antivirals, virus clearance in surviving in vitro plants was determined by RT-(q)PCR in samples aseptically collected from shoot tips. The pathogen-negative shoot tips were further subcultured on antiviral-free media at four-week intervals and retested 7–11 months later to confirm the successful elimination of pathogens.

### 2.4. Plant Material & RNA Extraction

A sample for RNA extraction was obtained by taking shoot tips along with several leaves, with a combined weight of up to 100 mg. The samples were processed with a GeneJET Plant RNA Purification Kit (Thermo Fisher Scientific, Waltham, MA, USA), and the extracted RNA was quantified and quality-controlled using a NanoDrop ND-1000 spectrophotometer (Thermo Fisher Scientific).

### 2.5. Diagnostics of SnIV-1 and AHVd by RT-PCR

Each RNA extract, amounting to half a microgram, was subjected to reverse transcription by a RevertAid First Strand cDNA Synthesis Kit (Thermo Fisher Scientific) with random primers according to the manufacturer’s instructions. PCR (Table 1) was performed using either Phire Hot Start II DNA Polymerase (Thermo Fisher Scientific) or PPP Master Mix (Top-Bio, Vestec, Czech Republic).

The *Malus domestica* NADH dehydrogenase’s mRNA was used as an internal control for RNA extraction and cDNA amplification. The PCR products were analysed by agarose gel electrophoresis. Selected products were directly Sanger sequenced. If the direct sequencing was of poor quality, then the product was cloned into pJET1.2 cloning vector (Thermo Fisher Scientific), and the resulting pDNA constructs were sequenced. All obtained sequences were deposited into the NCBI GenBank (cultivar ‘Jonagored Supra’: AHVd—ON564295, SnIV-1—OR137985; cultivar ‘Selena’: AHVd—ON564296, SnIV-1— OR137986; cultivar ‘Šampion’: AHVd—ON564299).

### 2.6. Virus and Viroid Diagnostics by RT-qPCR

All RT-qPCR assays were carried out on a CFX96 real-time PCR detection system (Bio-Rad, Hercules, CA, USA). The 10 µL reaction was prepared from 5 µL of the tenfold diluted cDNA, 0.25 µL of forward and reverse primers (final concentration 250 nM, Table 1), 2.75 μL of nuclease-free water, and 2 µL of 5 HOT FIREPol EvaGreen qPCR Mix Plus (Solis BioDyne, Taru, Estonia).

Reaction conditions were set up with a three-step cycling protocol—95 °C for 12 min, followed by 40 cycles of 95 °C for 10 s, 60 °C for 20 s, and 72 °C for 20 s. Dissociation curve analysis was performed by ramping from 65 °C to 95 °C in 0.5 °C increments for 5 s to verify the specificity of primer amplification and the presence of potential primer dimers based on the presence of a single peak. No-template and positive controls were included to check for potential cross-contamination and the presence of genomic DNA. The NADH mRNA was used as an internal endogenous control (Table 1). Data were analysed using the Bio-Rad CFX Maestro 1.1 software (Bio-Rad) and the R software version 4.1.0 under RStudio version 2021.09.1+372.

## 3. Results

The results regarding antiviral phytotoxicity and pathogen elimination are summarized in Figure 3.

All shoot tips treated with ribavirin declined at concentrations of 320 mg L^−1^ and higher. The 80 mg L^−1^ concentration was also highly phytotoxic, with only 5 shoot tips surviving out of a total of 30 treated. However, the concentration 160 mg L^−1^ was not as lethal, as 19 shoot tips out of 30 tips survived the treatment.

All shoot tips treated with rimantadine died at concentrations of 320 mg L^−1^ and higher. There was no visible phytotoxic effect of zidovudine on the shoot growth of cultures treated in vitro on media over the entire concentration range in all cultivars. There was no clear difference in the sensitivity of the three cultivars used in the experiments toward individual antivirals.

### 3.1. Elimination of SnIV-1

**Figure 2:**
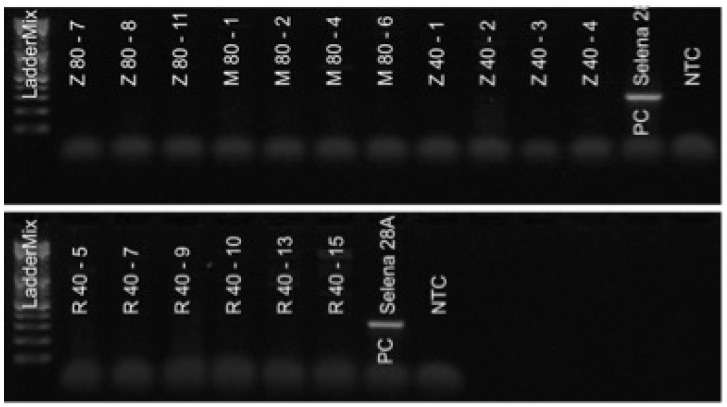
Results of RT-PCR assay for SnIV-1. The original Selena material was used as the positive control (PC). NTC—no template control, R—ribavirin, M—rimantadine, Z—zidovudine, followed by concentration in mg L^−1^ and mericlone number.

Ribavirin, rimantadine, and zidovudine eliminated SnIV-1 in both ‘Selena’ and ‘Jonagored Supra’. An example of an agarose gel with results for the cultivar ‘Selena’ is shown in Figure 2. RT-PCR was used at the beginning but was replaced by RT-qPCR (Table 1) due to the latter’s reduced processing time and inherent ability to prevent contamination of the laboratory environment with amplicon products.

**Figure 3:**
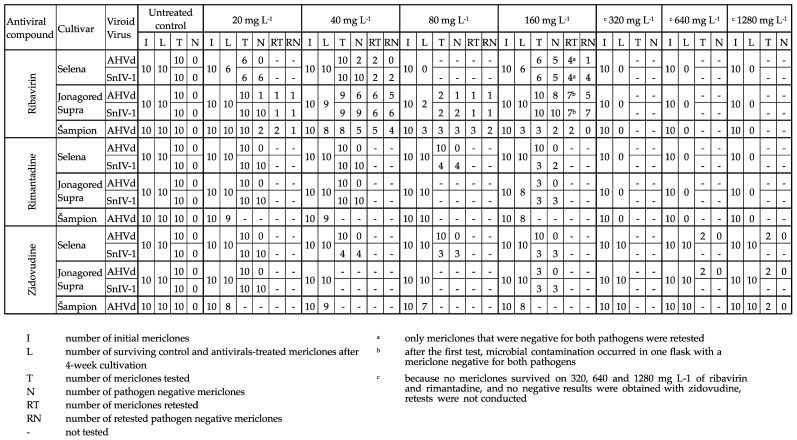
Results of phytotoxicity and efficiency of the antivirals.

For all the antivirotics, the effective concentration was only 20 mg L^−1^ (Figure 3), with a sanitation efficiency of 98% among all tested explants. Validation of SnIV-1 elimination by retesting was performed only on shoot tips that were also negative for AHVd, following the aim of the study. In addition, control testing of mixed samples of shoot tips that were not treated with antivirals, performed at the end of the project in 2023, confirmed also the elimination of SnIV-1 by passaging alone.

### 3.2. Elimination of AHVd

Elimination of AHVd from all apple cultivars was possible, although it was more difficult than elimination of SnIV-1. Ribavirin was effective at concentrations of 20, 40, and 80 mg L^−1^ in the cultivar ‘Šampion’, at concentrations of 40 and 160 mg L^−1^ in the cultivar ‘Selena’, and at concentrations of 20, 40, 80, and 160 mg L^−1^ in the cultivar ‘Jonagored Supra’, while rimantadine and zidovudine were not effective at all (Figure 3).

We calculated the efficiency of ribavirin for AHVd elimination as a proportion of the mericlones that tested negative even after the retest to all mericlones that survived ribavirin chemotherapy. The sanitation efficiency was 26% in the entire concentration range; therefore, it was possible to obtain plants that were negative for SnIV-1 and AHVd after they had been detected in initial plants.

## 4. Discussion

Apple hammerhead viroid (AHVd, *Pelamoviroid*; family *Avsunviroidae* [11]) has been identified in apple trees and is associated with symptoms such as trunk splitting, mosaic, necrosis, shoot decline, and dieback in China [28]. This problem has also been reported in various apple cultivars in Canada [13], the United States, Japan, Italy, Spain, New Zealand [29,30], Republic of Korea [31], India [32], Tunisia [33], the Czech Republic, and Hungary [10]. These data indicate that AHVd is widespread worldwide, and further research is needed to test methods for its elimination from apple propagation material. The elimination of viroids from infected plants has been a challenge for the past several decades, and various approaches such as thermotherapy, cold therapy, tissue culture, in vitro micrografting, and cryotherapy have been tested in attempts to cure plant tissues from viroid infection [34]. While shoot tip culture in combination with cold or heat therapy [35,36] has been successfully used to combat the apple scar skin viroid, the literature on the use of chemotherapy to eliminate viroids from apple is scarce [37].

The concentration range of antivirals in our experiments was set from 20 mg L^−1^ up to 1280 mg L^−1^ to account for the potential phytotoxicity of rimantadine and zidovudine, which had not yet been tested in apple cultivars. The number of surviving mericlones decreased with increasing concentrations of ribavirin and rimantadine. However, in the case of ribavirin, the observed phytotoxicity of 160 mg L^−1^ was unexpectedly low compared to 80 mg L^−1^. Based on the eradication results, we hypothesize that at 160 mg L^−1^, the elimination of AHVd reached its highest efficiency. Therefore, the phytotoxic effect for a higher number of clones may have been outweighed by the benefits of the viroid replication suppression and decrease of the viroid RNA concentration. The difference between the number of surviving and tested mericlones in Figure 3 resulted from the death of some mericlones due to microbial contamination during their serial passaging. Based on the visual appearance of the contamination, we assume that it was an internal bacterial infection that did not significantly manifest after sterilization in the initial initiation phase of the in vitro culture or during the subsequent multiplication and chemotherapy. Combined with the stress of chemotherapy, the plants may have been weakened and become more susceptible to infection.

Because no explant of any cultivar treated with ribavirin at a concentration higher than 160 mg L^−1^ survived, and no elimination of AHVd was observed with rimantadine and zidovudine, in view of the goal of our study to obtain pathogen-free cultivars, we focused our next efforts on the retesting of AHVd-negative mericlones. Consequently, we tested only a few mericlones that survived 320, 640, and 1680 mg L^−1^ concentrations of rimantadine or zidovudine, but all results returned positive. Retests for AHVd were carried out after 7–11 months and will be repeated during weaning and multiplication of the plants. The recommended interval in the literature for verifying the elimination of virus-like pathogens is at least 4–6 months [5].

A recent review by Wang et al. [37] indicates that apple-infecting viroids are difficult to eliminate by shoot therapy [36] or chemotherapy alone [38], and that a combination of thermotherapy [39] or chemotherapy [16] with shoot tip culture has been proven to be much more efficient for plant pathogen eradication. Bettoni et al. [5] achieved 25–75% efficiency of AHVd elimination from in vitro apple rootstocks by thermotherapy combined with cryotherapy. However, our results show the use of single chemotherapy resulting in 26% elimination of AHVd by ribavirin. In contrast to Hu et al. [38], who found ribavirin inefficient in eliminating apple scar skin viroid from apple plants, our results provide good evidence that ribavirin can be effectively used to eliminate of AHVd from apple plants.

SnIV-1 was first described in wild *Solanum nigrum* plants in France by Ma et al. [12]. Its first detection in apple was reported from a survey in Okanagan Valley orchards in British Columbia, Canada, by Xiao et al. [6], who identified several SnIV-1 sequences during HTS testing of declining trees. Their sequences MN216370.1, MN216373.1, and MN216376.1 show 97–98% identity with ours. Our detection of SnIV-1 in apple is the first in Europe. The natural host range of SnIV-1 may be broad and include both woody and herbaceous hosts, as there is an SnIV-1 isolate hosted by *Physalis* sp. in the GenBank database (accession numbers OL472060-OL472062).

The successful elimination of SnIV-1 ilarvirus from apple cultivars by zidovudine and rimantadine in our experiments agrees well with the results of Pavelková et al. [18] and Kudělková et al. [17]. Pavelková et al. [18] successfully used zidovudine for 100% elimination of prune dwarf virus (PDV), Prunus necrotic ringspot virus (PNRSV), two ilarviruses taxonomically related to SnIV-1, and plum pox virus (PPV; a potyvirus) from peach trees of the cultivar ‘Red Haven’ at concentrations of 25 mg L^−1^ and 50 mg L^−1^ with higher efficiency than ribavirin at 20 mg L^−1^, which was used as the reference. Kudělková et al. [17] found that rimantadine at concentrations of 25 and 50 mg L^−1^ was as effective as ribavirin at the same concentrations for the 80–90% elimination of PDV, PNRSV, and PPV from infected peach cultivars. Because SnIV-1 is a novel virus that has only recently been found to infect apple [6], no data are available on attempts to eliminate it from apple or other cultivated crops at all. However, the high percentage of SnIV-1 elimination by ribavirin is comparable to the elimination of other ilarviruses in apple [18]. Further studies are necessary to fully understand the efficiency of SnIV-1 eradication through tip culturing, which has shown promise in effectively eradicating other virus–plant systems [40].

## 5. Conclusions

Our results showed that two recently discovered apple pathogens, AHVd and SnIV-1, could be eliminated from infected material by a combination of in vitro shoot culture and chemotherapy with antivirals. These data might be practically useful for laboratories lacking expensive equipment and training for cryotherapy. The efficiency of rimantadine and zidovudine for in vitro plant sanitation, reported in other authors’ experiments, was not confirmed for AHVd elimination in our work. Following the promising results of ribavirin chemotherapy, we plan to utilize more antivirotics that can act as nucleoside analogues.

## Figures and Tables

**Table 1:** Sequences of the PCR primers used in the study.

Target	Primer Name	Primer Sequence (5’-3’)	Usage; Region	Reference
*Malus* *domestica*, NADH ^a^	2277	TGCTCCATGGATCTCATCGG	RT-qPCR, RT-PCR, RNA extraction and PCR amplification control; protein-coding region	[10]
	2278	AATCGAGGGCTATGCGGATC		
AHVd ^b^	2115	CTGCCGAAACAGAGGTTGGA	RT-qPCR, RT-PCR; genome	
	2116	GAGAAGTCGCTCTCTCTCGC		
SnIV-1 ^c^	2696	TTTGGGTTTGTAGCCGAATC	RT-qPCR; gene for coat protein	this work
	2697	TTACCTCCGAGATCAACGTC		
	2365	GCCCTATCTCTACCCGAGGT	RT-PCR; gene for polymerase	
	2363	TGTCGAAGGACAGCCGAAAT		

^a^ NADH dehydrogenase’s mRNA, ^b^ apple hammerhead viroid, ^c^ Solanum nigrum ilarvirus.

## Data Availability

Sequence data are available on GenBank under the following accession numbers: ON564295, OR137985, ON564296, OR137986, and ON564299.

## References

[1] Loebenstein G., van Regenmortel M., Mahy B. (2009). Plant virus diseases: Economic aspects. Desk Encyclopedia of Plant and Fungal Virology.

[2] Jones R.A.C., Naidu R.A. (2019). Global Dimensions of Plant Virus Diseases: Current Status and Future Perspectives. Annu. Rev. Virol..

[3] Hilaire J., Tindale S., Jones G., Pingarron-Cardenas G., Bačnik K., Ojo M., Frewer L.J. (2022). Risk perception associated with an emerging agri-food risk in Europe: Plant viruses in agriculture. Agric. Food Secur..

[4] Pautasso M., Petter F., Rortais A., Roy A.-S. (2015). Emerging risks to plant health: A European perspective. CAB Rev..

[5] Bettoni J.C., Fazio G., Costa L.C., Hurtado-Gonzales O.P.P., Al Rwahnih M., Nedrow A., Volk G.M.M. (2022). Thermotherapy Followed by Shoot Tip Cryotherapy Eradicates Latent Viruses and Apple Hammerhead Viroid from In Vitro Apple Rootstocks. Plants.

[6] Xiao H., Hao W., Storoschuk G., MacDonald J.L., Sanfaçon H. (2022). Characterizing the Virome of Apple Orchards Affected by Rapid Decline in the Okanagan and Similkameen Valleys of British Columbia (Canada). Pathogens.

[7] Boonham N., Kreuze J., Winter S., van der Vlugt R., Bergervoet J., Tomlinson J., Mumford R. (2014). Methods in virus diagnostics: From ELISA to next generation sequencing. Virus Res..

[8] Patel R., Mitra B., Vinchurkar M., Adami A., Patkar R., Giacomozzi F., Lorenzelli L., Baghini M.S. (2022). A review of recent advances in plant-pathogen detection systems. Heliyon.

[9] Massart S., Candresse T., Gil J., Lacomme C., Predajna L., Ravnikar M., Reynard J.S., Rumbou A., Saldarelli P., Skoric D. (2017). A Framework for the Evaluation of Biosecurity, Commercial, Regulatory, and Scientific Impacts of Plant Viruses and Viroids Identified by NGS Technologies. Front. Microbiol..

[10] Varallyay E., Pribylova J., Galbacs Z.N., Jahan A., Varga T., Spak J., Lenz O., Franova J., Sedlak J., Koloniuk I. (2022). Detection of Apple Hammerhead Viroid, Apple Luteovirus 1 and Citrus Concave Gum-Associated Virus in Apple Propagation Materials and Orchards in the Czech Republic and Hungary. Viruses.

[11] Serra P., Messmer A., Sanderson D., James D., Flores R. (2018). Apple hammerhead viroid-like RNA is a bona fide viroid: Autonomous replication and structural features support its inclusion as a new member in the genus Pelamoviroid. Virus Res..

[12] Ma Y.X., Marais A., Lefebvre M., Faure C., Candresse T. (2020). Metagenomic analysis of virome cross-talk between cultivated Solanum lycopersicum and wild Solanum nigrum. Virology.

[13] Messmer A., Sanderson D., Braun G., Serra P., Flores R., James D. (2017). Molecular and phylogenetic identification of unique isolates of hammerhead viroid-like RNA from ‘Pacific Gala’ apple (*Malus domestica*) in Canada. Can. J. Plant Pathol..

[14] Graci J.D., Cameron C.E. (2006). Mechanisms of action of ribavirin against distinct viruses. Rev. Med. Virol..

[15] Hansen A.J. (1989). Antiviral chemicals for plant-disease control. Crit. Rev. Plant Sci..

[16] Hu G.J., Dong Y.F., Zhang Z.P., Fan X.D., Ren F., Zhou J. (2015). Virus elimination from in vitro apple by thermotherapy combined with chemotherapy. Plant Cell Tissue Organ Cult..

[17] Kudelkova M., Pavelkova R., Ondrusikova E. (2017). Virus elimination in peach using chemotherapy. Acta Hortic..

[18] Pavelkova R., Kudelkova M., Ondrusikova E., Eichmeier A. (2015). Virus Elimination in Peach cv. ‘Red Haven’ by Chemotherapy. Agric. Commun..

[19] Murashige T., Skoog F. (1962). A Revised Medium for Rapid Growth and Bio Assays with Tobacco Tissue Cultures. Physiol. Plant..

[20] Sidwell R.W., Witkowski J.T., Allen L.B., Robins R.K., Khare G.P., Huffman J.H. (1972). Broad-spectrum antiviral activity of Virazole: 1-beta-D-ribofuranosyl-1,2,4-triazole-3-carboxamide. Science.

[21] Huffman J.H., Sidwell R.W., Khare G.P., Witkowski J.T., Allen L.B., Robins R.K. (1973). In vitro effect of 1-beta-D-ribofuranosyl-1,2,4-triazole-3-carboxamide (virazole, ICN 1229) on deoxyribonucleic acid and ribonucleic-acid viruses. Antimicrob. Agents Chemother..

[22] James D. (2001). Long term assessment of the effects of in vitro chemotherapy as a tool for apple stem grooving virus elimination. Acta Hortic..

[23] Wright K. (1986). AIDS therapy. First tentative signs of therapeutic promise. Nature.

[24] Tochikura T.S., Nakashima H., Yamamoto N. (1989). Antiviral agents with activity against human retroviruses. J. Acquir. Immune Defic. Syndr. Hum. Retrovirol..

[25] Hannoun (1988). Rimantadine in the prevention and treatment of influenza A. Rev. Médecine Interne.

[26] Thomaston J.L., Samways M.L., Konstantinidi A., Ma C., Hu Y., Macdonald H.E.B., Wang J., Essex J.W., DeGrado W.F., Kolocouris A. (2021). Rimantadine binds to and inhibits the influenza A M2 proton channel without enantiomeric specificity. Biochemistry.

[27] Sasaki-Tanaka R., Shibata T., Moriyama M., Okamoto H., Kogure H., Kanda T. (2022). Amantadine and Rimantadine Inhibit Hepatitis A Virus Replication through the Induction of Autophagy. J. Virol..

[28] Zhang Z.X., Qi S.S., Tang N., Zhang X.X., Chen S.S., Zhu P.F., Ma L., Cheng J.P., Xu Y., Lu M.G. (2014). Discovery of Replicating Circular RNAs by RNA-Seq and Computational Algorithms. PLoS Pathog..

[29] Szostek S.A., Wright A.A., Harper S.J. (2018). First Report of Apple Hammerhead Viroid in the United States, Japan, Italy, Spain, and New Zealand. Plant Dis..

[30] Wright A.A., Cross A.R., Harper S.J. (2020). A bushel of viruses: Identification of seventeen novel putative viruses by RNA-seq in six apple trees. PLoS ONE.

[31] Lim S., Moon J.S., Cho I.S., Kim H.R., Lee S.H. (2019). First Report of Apple Hammerhead Viroid Infecting Apple Trees in South Korea. Plant Dis..

[32] Nabi S.U., Baranwal V.K. (2020). First Report of Apple Hammerhead Viroid Infecting Apple Cultivars in India. Plant Dis..

[33] Hamdi I., Soltani R., Baraket G., Varsani A., Najar A. (2022). First report of apple hammerhead viroid infecting ‘Richared Delicious’ apple (*Malus domestica*) in Tunisia. J. Plant Pathol..

[34] Barba M., Hosakawa M., Wang Q.-C., Taglienti A., Hamborg Z., Hadidi A., Flores R., Randles J.W., Palukaitis P. (2017). Viroid Elimination by Thermotherapy, Cold Therapy, Tissue Culture, In Vitro Micrografting, or Cryotherapy. Viroids and Satellites.

[35] Desvignes J.C., Grasseau N., Boye R., Cornaggia D., Aparicio F., Di Serio F., Flores R. (1999). Biological properties of apple scar skin viroid: Isolates, host range, different sensitivity of apple cultivars, elimination, and natural transmission. Plant Dis..

[36] Hu G.J., Zhang Z.P., Dong Y.F., Fan X.D., Ren F., Zhu H.J. (2015). Efficiency of virus elimination from potted apple plants by thermotherapy coupled with shoot-tip grafting. Austral. Plant Pathol..

[37] Wang M.-R., Bi W.-L., Bettoni J.C., Zhang D., Volk G.M., Wang Q.-C. (2022). Shoot tip cryotherapy for plant pathogen eradication. Plant Pathol..

[38] Hu G.J., Dong Y.F., Zhang Z.P., Fan X.D., Ren F. (2022). Inefficiency of ribavirin to eliminate apple scar skin viroid from apple plants. Plant Cell Tissue Organ Cult..

[39] Hu G.J., Dong Y.F., Zhang Z.P., Fan X.D., Ren F., Li Z.N. (2017). Efficacy of virus elimination from apple by thermotherapy coupled with in vivo shoot-tip grafting and in vitro meristem culture. J. Phytopathol..

[40] Bhat A.I., Rao G.P. (2020). Virus Elimination by Meristem-Tip Culture. Characterization of Plant Viruses.

